# Single port access sleeve gastrectomy: Is it reasonable?

**DOI:** 10.4103/0972-9941.78354

**Published:** 2011

**Authors:** Ramon Vilallonga, Josep Rius, José Manuel Fort, Manuel Armengol

**Affiliations:** Department of General Surgery, Endocrine, Bariatric and Metabolic Unit, Universitary Hospital Vall d’Hebron, Barcelona, Spain; 1 Bariatric Surgery Unit, Hospital USP La Colina, Santa Cruz de Tenerife, Spain

**Keywords:** Morbid obesity, sleeve gastrectomy, single port access, transumbilical access

## Abstract

This short letter is in response to the article published in your publication about single-incision laparoscopic bariatric surgery, by Chih-Kun Huang. We want to focus on the technical aspects.

This personal view point is in response to the recent article published in your Journal about single-incision laparoscopic bariatric surgery, by Chih-Kun Huang.[1] We have also performed single-incision laparoscopic sleeve gastrectomy. For this, we used trocar (GelPOINT^®^ advanced access platform), standard straight graspers and multiple applications of a 60-mm stapler (Echelon Flex-Ethicon-Endosurgery). A 5mm extra-large harmonic ACE 36cm curved shears (Ethicon) was used in this surgery. The beginning of the sleeve was started 5 cm from the pylorus. From our point of view, the most important and challenging moment of surgery was during the dissection of the left crus, with fundus mobilization from the short splenic vessels. At this stage of the procedure, the liver could obscure the view and correct counter traction may be difficult. According to our standard techniques, in standard laparoscopic sleeve gastrectomy, a continuous polypropylene suture (3 / 0) (Prolene^®^, Ethicon-Endosurgery) was performed throughout the vertical border of the sleeve, with a needle holder and grasper, through the Single Port Access (SPA) trocar. For this manoeuvre, an extra 5 mm trocar was used to pull the liver up in the upper part. Also this trocar was used to place the final drain. This trocar was only placed and used when performing the continuous polipropliene suture.

As we already know, laparoscopic sleeve gastrectomy has been recognised since a few years as a valid bariatric procedure, which has shown good results for reduction of weight and co-morbidities, in obese patients.[2] It also appears that no anastomoses are required, which makes this procedure attractive to surgeons. However, an accurate technique must be used in order to avoid any serious complications, such as fistula or leakage. Many authors have considered performing sleeve gastrectomy entirely through the umbilicus, as it is safe, technically feasible and reproducible.[3]

From our bariatric experience, an important detail to be taken in account is the need for performing a continuous suture of the gastric border. Many surgeons use tissue reinforcement for the staple line, such as GORE^®^ (SEAMGUARD^®^) or Duet Trs™ (Covidien™), which offer bioabsorbable staple line reinforcement, specifically engineered to reduce the incidence of perioperative leaks and bleeding in a variety of open and minimally invasive surgeries. The use of these materials with the staple line offer saving of time and extra trocar placement, and it also avoids undertaking a continuous suture through an SPA. However, it is still a controversial issue in sleeve gastrectomy.[4]

The SPA approach is feasible with standard and slightly modified instruments for standard sleeve gastrectomy, thus posing a minimal additional challenge to the laparoscopic surgeon. Accordingly, we believe that only trained bariatric surgeons should use this approach for sleeve gastrectomy. An extra trocar should be used during the initial experience of single incision sleeve gasterctomy. For these reasons, it seems reasonable to assume that this technique is feasible and that new devices such as tissue reinforcements can make this approach easier and more feasible. However, this will have to be evaluated with other studies in order to determine if the new approach means new technical aspects and increased risk of leakage or fistula of the sleeve. New approaches now described, such as the transvaginal sleeve gastrectomy, will have to be compared.[5]

In our opinion, this novel approach is feasible, but not always easily reproducible according to our own standard techniques.
